# Therapy resistance and metastasis

**DOI:** 10.1007/s10585-023-10205-x

**Published:** 2023-03-23

**Authors:** Jonathan P. Sleeman

**Affiliations:** 1 Medical Faculty Mannheim, University of Heidelberg, ECAS, Ludolf-Krehl-Str. 13 – 17, 68167 Mannheim, Germany; 2 Karlsruhe Institute for Technology (KIT), IBCS-BIP, Campus Nord, 76344 Eggenstein-Leopoldshafen, Germany

**Keywords:** Metastasis, Targeted therapy, Immune therapy, Microenvironment, Therapy resistance

Recent years have witnessed dramatic advances in the efficacy of cancer treatments. As a consequence, the prognosis for those diagnosed with a number of different types of cancer has improved significantly. Progress in many areas has contributed to this success. Technological developments such as artificial intelligence and molecular imaging have improved surgical procedures and the precision of radio-oncological interventions [1]. The introduction of neoadjuvant therapy for many types of cancer allows occult metastasis to be treated as early as possible [2]. The most dramatic improvements in anti-cancer treatment have arguably come through the implementation of targeted therapies built on years of careful and painstaking molecular analysis of tumorigenesis and progression, and more recently through immune checkpoint therapies, reflecting the increasing appreciation of the role that the tumor microenvironment plays in oncology and its potential as a therapeutic target. As a consequence, cancers such as melanoma, which only a couple of decades ago were considered to almost invariably result in the rapid demise of the patient, can now be kept in check over long periods [3]. These developments are similarly allowing increasing numbers of patients suffering from the six most common types of cancer to live with metastatic disease for longer periods of time [4].

While cancers such as pancreatic carcinoma and lung malignancies still represent a considerable challenge, the ability of modern oncological intervention strategies to keep metastatic disease under control for an increasing number of cancer types raises the prospect that metastatic cancer may in the future be rendered a chronic disease for many patients, rather than representing an inevitable death sentence [5]. This prospect turns the attention to the reason why metastatic disease is not controllable in some patients, and why therapies that are initially able to restrict the growth and progression of metastatic disease subsequently become ineffective, leading to the demise of the patient. Thus, therapy resistance is increasingly being seen as a major obstacle to the objective of rendering stage IV cancer a chronic disease [6]. Resistance may be intrinsic to a particular type of cancer or at the level of individual patients, or may be acquired through adaptive mechanisms such as the induction of a drug tolerant persister phenotype, and subsequent resistance-endowing mutations [7].

Much remains to be understood about the mechanisms of therapy resistance, and progress in this area will be key to identifying ways of overcoming therapy failure for metastatic cancer. In recognition of the importance of this research field for cancer patients with stage IV disease, Clinical & Experimental Metastasis has introduced a new collection entitled “Mechanisms of resistance to cancer therapy”. Recent contributions to this collection include reviews covering the resistance mechanisms to targeted therapy [8], and the importance of precision oncology for overcoming resistance [9]. The journal has set itself the objective of striving to support rapid developments in this field, and readers are warmly encouraged to contribute manuscripts to this important area of metastasis research as part of this effort.
